# Automatic detection of cognitive events using machine learning and understanding models’ interpretations of human cognition

**DOI:** 10.1038/s41598-025-16165-4

**Published:** 2025-08-20

**Authors:** Quang Dang, Murat Kucukosmanoglu, Michael Anoruo, Golshan Kargosha, Sarah Conklin, Justin Brooks

**Affiliations:** 1 Department of Computer Science and Electrical Engineering, University of Maryland, Baltimore County, Baltimore, MD 21250 USA; 2 D-Prime LLC, McLean, VA 22101 USA; 3 Center for Women’s Biobehavioral Health Research, Department of Psychiatry, University of Pittsburgh Medical Center, Pittsburgh, PA 15219 USA

**Keywords:** Cognitive events detection, Cognitive classification, Feature importance, Neural networks, Explainable AI, Machine learning, Deep learning, Human behaviour, Machine learning, Cognitive control, Computer science

## Abstract

The pupillary response is a valuable indicator of cognitive workload, capturing fluctuations in attention and arousal governed by the autonomic nervous system. Cognitive events, defined as the initiation of mental processes, are closely linked to cognitive workload as they trigger cognitive responses. In this study, we detect cognitive events for the task-evoked pupillary response across four domains (vigilance, emotion processing, numerical reasoning, and short-term memory). The problem is framed as a binary classification. We train one generalized model and four task-specific models on 1-s pupil diameter and gaze position segments. Five models achieve MCC between 0.43 and 0.75. We report three key findings: (1) the generalized model reduces the specificity to enhance the sensitivity, illustrating the trade-off from specialization to generalization; (2) the permutation feature importance analyses show that both pupil dilation and gaze position contribute to model predictions, with task-specific models focusing on task-specific structure patterns to predict while the generalized model is using human cognitive responses; and (3) in an online simulation environment, models performance decreases by approximately 0.05 on MCC. The findings highlight the potential of machine learning applied to pupillary signals for rapid, individualized detection of cognitive events.

## Introduction

Cognitive workload, the mental effort or cognitive demand associated with a specific task, constitutes a fundamental concept in human cognitive neuroscience. Interdisciplinary efforts to quantify cognitive workload in real time are of widespread interest with broad applications. It is a cornerstone concept in human cognitive neuroscience with widespread interest and diverse applications^1^. Understanding an individual’s cognitive workload provides insight into their mental state and cognitive status, enhancing personalized learning and real-time cognitive assessments. Innovative analytic tools and approaches such as machine learning have emerged for analyzing and estimating levels of cognitive workload from physiological signals obtained with electrophysiology^2^, electrocardiography^3,4^, electrodermal activity^5^, and eye tracking^6^.

While multiple researchers classify the cognitive workload using machine learning^7–9^, there is no universal agreement on a method for quantifying the threshold between low and high workload. The results can vary depending on the dataset and methodology used. For example, Gupta et al. introduce a method for estimating cognitive workload using EEG data, functional brain connectivity, and deep learning algorithms^2^. Their approach achieves a classification accuracy of 80.87%. In this paper, we focus on predicting cognitive events using task?evoked pupillary responses (TEPR) as the dataset. Although several studies predict cognitive events using machine learning approaches^10,11^, the majority of researchers rely on EEG data to make these predictions, commonly known as event?related potentials^12^. Multiple studies highlight the importance of TEPR in measuring cognitive workload^6,13,14^ as an index of psychophysiologic arousal. However, it remains an open question whether a machine learning approach can utilize the TEPR as a cognitive event detector (i.e. detecting the onset of a cognitive task stimulus)

TEPR is a change in pupil diameter that occurs in response to cognitive load. Evidence suggests that TEPR reflects the amount of cognitive effort required to perform the task. TEPR is observed during widespread cognitive processes^15^ and has been proposed to function as both a gauge and a filter to optimize cognitive functioning^16^. TEPR focuses on attention^17^, auditory discrimination^18^, mathematical problem solving, visual working memory, long-term memory tasks^19^, multiple object tracking^20^, decision-making^21^, naming tasks^22^, and vigilance^23^. The dynamic changes in TEPR serve as essential tools for understanding cognitive processes, individual differences in cognitive abilities, resource allocation, and preferences^24^.

In this paper, we aim to predict the onset of cognitive events that is the start of each TERP. While the majority of TEPR-related research focuses on estimating cognitive workload, only a limited number of studies investigate the prediction of cognitive event onset using TEPR data. Predicting cognitive events has several advantages over predicting cognitive workload. The cognitive workload requires a longer time to fully manifest as it reflects the mental effort required to complete a specific task. The prediction timeframe for workload varies based on the task’s duration and structure. In addition, the model typically predicts “low” or “high” states, which can be debatable to quantify the cognitive workload.

In contrast, cognitive events are the external load because they introduce new information that requires mental processing and response. Predicting cognitive events offers the advantage of using shorter data segments. The average time for the pupil to fully react to a cognitive event is about 0.6–0.75 s^25^, and our target window is 1 s. In this timeframe, the physiological signal required for recognizing the cognitive events is fully manifested. Another advantage of cognitive events is the availability of ground-truth data. In TEPR, stimulus onset times are the ground truth because they indicate the start of cognitive events. Our prediction task is similar to event-related potential. The distinction is that event-related potentials are acquired from the EEG signal and the event can be from the cognitive, sensory, or motor domain^12^. The cognitive events, in this paper, are limited to TERP and are measured exclusively through pupillary responses in TEPR.

Several physiological signals are widely used to study human cognition, including electrocardiograms (ECG), electroencephalograms (EEG), and pupillometry. However, ECG and EEG present limitations for the scope of this study. ECG requires a longer time frame to measure features, e.g. approximately 30 s to calculate heart rate and heart variability^26^, approximately 5 min to calculate the frequency domain of heart rate variability^27^. Our target window for cognitive events is less than 5 s. EEG signals have a low signal-to-noise ratio, and difficulty in interpreting^28^. EEG data collection equipment is often expensive, cumbersome, and invasive for daily life usage. In contrast, eye-tracking equipment provides a noninvasive method for real-time pupillary measurement, using screen-mounted or wearable devices. The pupillary signal enables analysis within shorter time windows, with as little as 1 s of data^29^, and can provide insights into neurocognitive processes by capturing gaze patterns, pupil dilation, and saccade eye movements^30^.

This study addresses two central research questions: (1) Can pupillary signals be used to predict cognitive events, and how well do such predictions generalize across different cognitive domains? (2) Does the machine learning model detect cognitive stimuli based on cognitive responses, or does it rely on unrelated features that do not reflect cognitive processing?

From an engineering perspective, we build machine learning models that take the pupillary signal (pupil diameter and gaze data) as inputs. The data comes from four TEPR tasks that sample four cognitive domains: vigilance, emotion processing, numerical processing, and short-term memory. We construct CNN models that can predict stimulus onset times, using 1-s segments of pupillary data. By translating the two research questions into two engineering objectives and adding one applied objective, we have three primary objectives for this study:

1. We construct five machine learning models: four task-specific models corresponding to four cognitive tasks, and one generalized model trained on the entire dataset. We evaluate the performance of these five models and analyze the trade-off between task-specific specialization and cross-task generalization.
2. We analyze the feature importance in each model. These features include human cognitive responses and unique task structures in each task. We evaluate the contribution of each feature to the model’s predictions.
3. We assess the models’ performance in an online environment to assess their usability in real-time scenarios. Analyzing the trade-offs between online and offline environments allows us to understand the usability and limitations of the models in practical settings.

## Results

### Exploratory data analysis

**Fig. 1:**
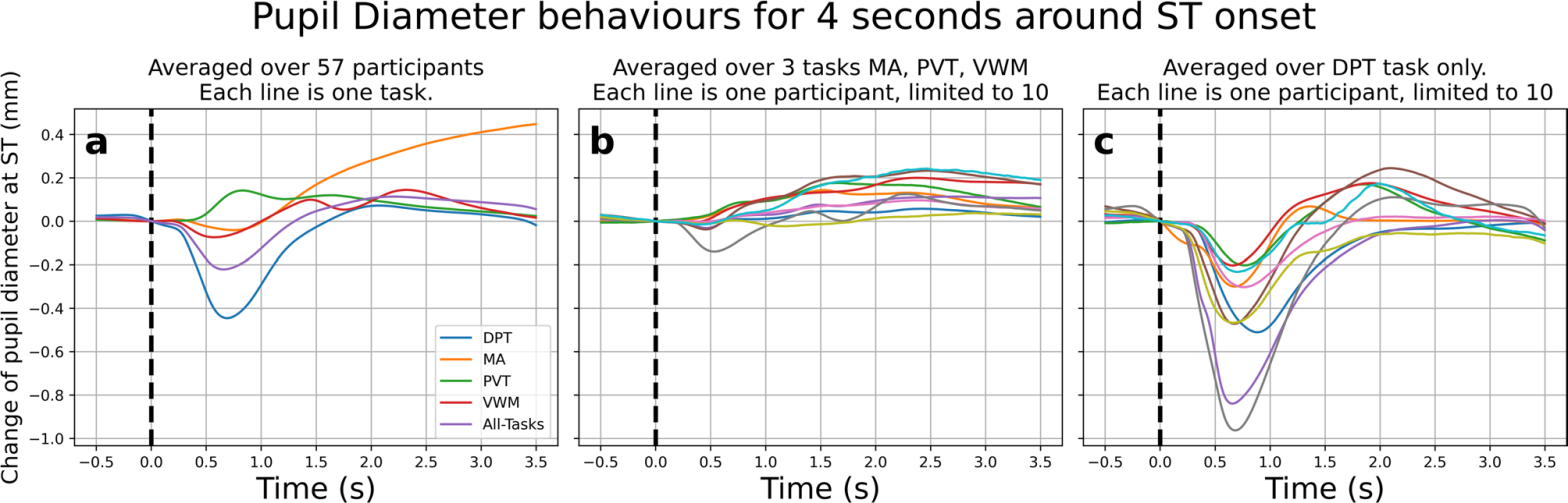
The mean pupil diameter changed after ST. The X-axis was time relative to the ST, while the Y-axis showed the change of the pupil diameter. Each line represented one task, and the “All-tasks” line showed the overall average for 4 tasks. Panel (**a**) averaged across 57 participants, panel (**b**) averaged across 3 tasks MA, PVT, and VWM, and panel (**c**) averaged for only the DPT task. In both panels (**b**) and (**c**), each line corresponded to one participant (color code shared). We limited 10 participants for display purposes.

This first section presented the exploratory data analysis. This section was necessary because some information would be needed for the sampling process in the “Methodology” and “Discussion” section. With more detail in the “Material” section, the dataset had four TEPR tasks: Dot Probe Task (DPT), Mental Arithmetic (MA), Psychomotor Vigilance Task (PVT), and Visual Working Memory (VWM). The stimulus onset time (ST) was the start of each trial. Focusing on pupil diameter, the majority of participants showed a median pupil diameter ranging from 3.5 to 4.5 mm with outliers, with outliers exceeding 5.5 mm or falling below 3 mm. The Fig. 1 showed the common pattern of pupil diameter response around ST.

In panel a of Fig. 1, all tasks except PVT showed an initial constriction lasting approximately 0.6 s, followed by dilation. By the end of this period, pupil diameter exceeded its initial size at ST, reflecting increased cognitive workload and sympathetic activation in response to new information^31,32^.

The panel b showed the average response for ten participants on MA, PVT, and VWM tasks while the panel c showed the average response for DPT exclusively. We limited the visualization to the 10 participants with the most available data for display purposes. In DPT tasks, the pupil diameter constricted significantly compared to the other three tasks, attributed to the pupillary light reflex (PLR). In the DPT task, the screen displayed before the ST was relatively dim compared to when the ST was presented, resulting in a sudden increase in brightness, and triggering the PLR. This response accounted for most of the observed pupil constriction during the DPT task. After re-dilation, pupil diameter in some participants exceeded baseline levels, indicating cognitive workload similar to the other tasks.

**Fig. 2:**
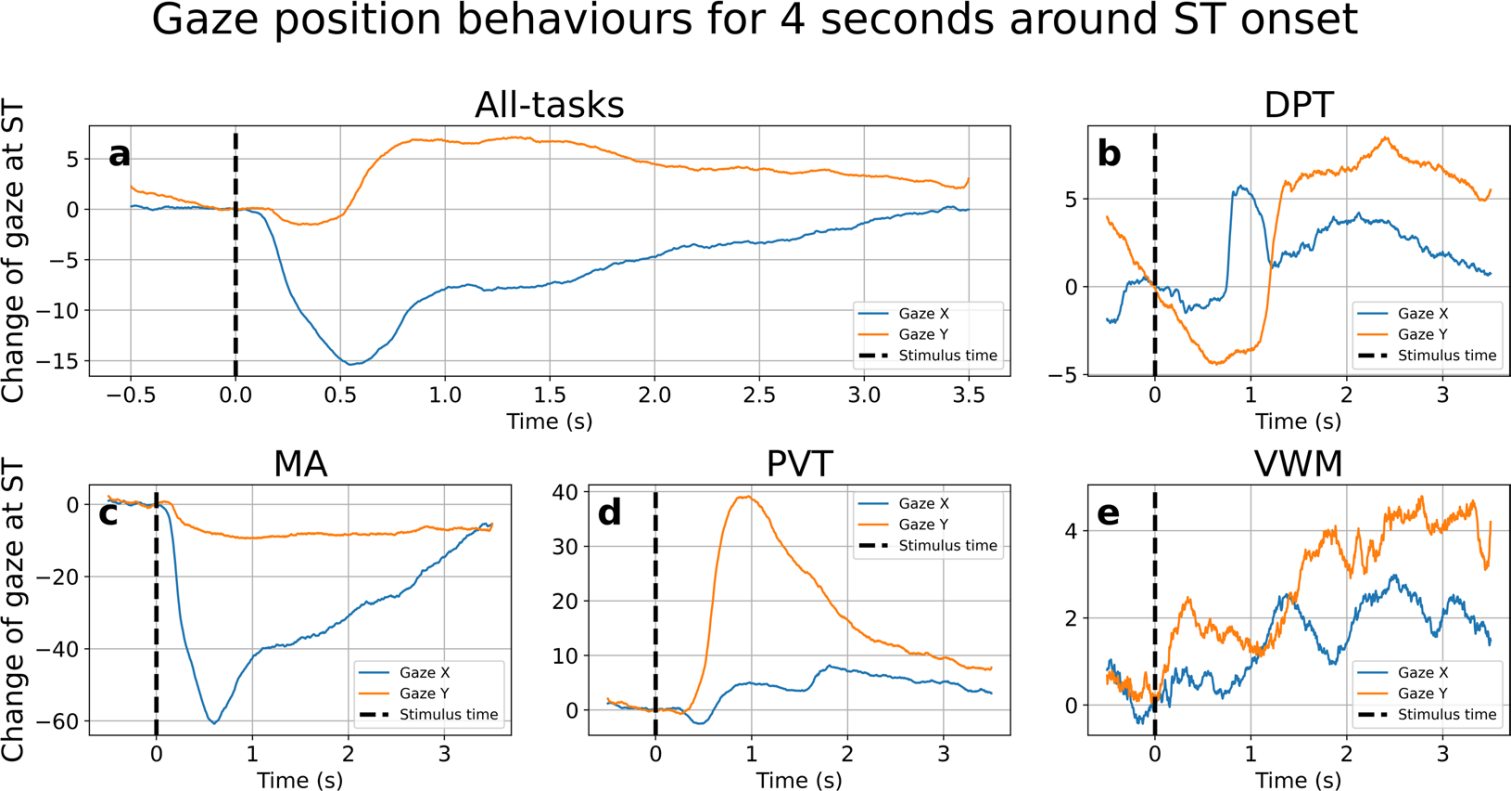
Mean gaze position changed after ST for each task. Panels for DPT, PVT, MA, and VWM displayed task-specific, while the “All-tasks” panel showed the average across all four tasks. The X-axis represented time relative to ST, and the Y-axis indicated the difference between gaze position at ST and at corresponding time points..

Moving on gaze position, Fig. 2 showed the average gaze position data for all participants using the same processing method as Fig. 1. The unit was screen coordinates, where (0,0) represented the top-left corner and (1000,1000) indicated the bottom-right corner. The average values for Gaze X and Gaze Y were − 1.53 and 4.18, respectively; the average standard deviations for Gaze X was 77 and for Gaze Y was 60, respectively.

Although the DPT tasks showed minimal eye movement within the time of interest, the MA and PVT tasks displayed saccadic eye movements between 0 and 0.5 s after ST, reflecting participants’ rapid gaze shifts toward new visual information. This shifting focus took about 0.5 s before the participant began processing the information presented.

In the VWM tasks, after averaging, we observed high-frequency signals. The high frequencies were not due to noise but attributable to the multiple patterns that were caused by the structure of the VWM tasks. Unlike the other tasks, where participants focused on a specific point of interest after ST, the VWM tasks had varying difficulty levels, ranging from easy (one image) to hard (six images). This led to diverse gaze patterns and a lack of a consistent trend across the tasks.

### Machine learning architecture comparison

Table 1 showed the comparison of 4 architectures: convolution neural network (CNN), bidirectional long short-term memory (BiLSTM), simple recurrent neural network (RNN), and multilayer perceptron (MLP). We used three evaluation metrics: accuracy (ACC), F1 score (F1), and Matthews Correlation Coefficient (MCC), with MCC as the primary metric. As mentioned in the “Sampling method” section, we framed the problem as binary classification where the “0” sample indicated the absence of ST and the “1” sample indicated the presence of ST. Because the dataset was imbalanced in favor of the “0” samples, we applied the Synthetic Minority Oversampling Technique (SMOTE) technique to re-balance the class sample population. The SMOTE technique was applied to only the training set, but not to the testing set. As a result, during training, five models saw an equal number of “0” and “1” samples. Therefore, during the prediction, there was no statistical bias between the population between the “0” and “1” classes. Four task-specific models (“DPT”, “MA”, “PVT”, “VWM”) were trained and tested on their respective tasks, while the “All-task” model was trained and tested on the entire dataset.

**Table 1:** Machine learning architecture comparison and result.

		CNN			BiLSTM			RNN			MLP		
		ACC	F1	MCC	ACC	F1	MCC	ACC	F1	MCC	ACC	F1	MCC
Model	“All-task”	0.78	0.73	0.55	0.78	0.73	0.55	0.62	0.54	0.22	0.77	0.73	0.54
	“DPT”	0.88	0.83	0.75	0.89	0.83	0.75	0.64	0.55	0.26	0.87	0.82	0.72
	“MA”	0.88	0.72	0.65	0.81	0.64	0.56	0.72	0.53	0.43	0.85	0.67	0.60
	“PVT”	0.84	0.70	0.59	0.82	0.71	0.60	0.65	0.57	0.39	0.84	0.72	0.60
	“VWM”	0.73	0.56	0.43	0.66	0.53	0.41	0.60	0.49	0.35	0.75	0.58	0.46

The results showed that the RNN architecture underperformed relative to the others. The other three architectures had similar performance levels within the margin of error. Therefore, for the remainder of the paper, we will focus exclusively on the CNN architecture, because CNN achieved marginally better results and benefited from faster computation due to its inherent parallelism while the BiLSTM processed data sequentially, as each time step depended on its previous step.

### CNN model performance

When the performance of the five CNN models in Table 1, measured in MCC, the model ranking from worst to best was: “VWM”, “All-task”, “PVT”, “MA”, and “DPT” with the MCC of 0.43, 0.55, 0.59, 0.65, and 0.75 respectively. In general, the performance drop in the generalized “All-task” model compared to the specialized models (“VWM”, “MA”, “PVT”, and “DPT”) was expected. Four models, excluding “VWM”, achieved an MCC above 0.5, which was considered a good score. In Table 2, we further broke down the results into specificity and sensitivity metrics to reveal the trade-offs between generalization and specialization.

**Table 2:** Additional metric for five CNN models.

		Metrics			
		Specificity	Sensitivity	Pearson	McNemar P-value
Model	“All-task”	0.80	0.75	1.0	1.0
	“DPT”	0.91	0.84	0.88	< 0.001
	“MA”	0.95	0.65	0.69	< 0.001
	“PVT”	0.89	0.70	0.77	< 0.001
	“VWM”	0.93	0.43	0.65	< 0.001

In Table 2, the generalized “All-task” model had the lowest specificity (0.80) and the second highest sensitivity value (0.75), behind the “DPT” model. By adding a variety of task types, the sensitivity increased at the expense of specificity. This demonstrated the trade-off between specialization (single-task) and generalization (four-task).

We included the Pearson correlation coefficient and the p-value of the McNemar test to compare each specialized model against the generalized “All-task” model. The McNemar p-values for all four specialized models were less than 1E-3, indicating that their predictions were statistically distinguishable from the prediction of the generalized model. In addition, Pearson correlation coefficients showed that the predictions of the four specialized models were moderately to strongly correlated with the “All-task” model predictions. For the “All-task” model, both the Pearson correlation coefficient and the McNemar test p-value were 1.0 because of self-comparison.

The “VWM” model displayed the lowest performance with an MCC score of 0.43. Its specificity value was compatible with that of the other three specialized models (“MA”, “PVT”, and “DPT”). Its low performance was due to a high number of false negatives. In the “Discussion” section, we discussed how the difficulty in predicting “1” samples for the “VWM” model was likely caused by differences in activation regions within the human brain.

The next analysis examined the amount of data required to predict cognitive events. Table 3 showed the “All-task” model’s performance using a CNN architecture in four sample durations: 0.5 s, 1 s, 2 s, and 3  s. All other variables remained constant, except for the sample duration. The results showed that the windows of 0.5 s and 3 s underperformed. The 0.5-s window provided insufficient information for accurate prediction. The 3-s window included data that was not relevant (i.e. far distant from cognitive events), which introduced more noise than benefit to the model. The optimal window fell between 1 s and 2 s, with the 2-s duration marginally outperforming the 1-s duration. We chose 1-s windows as the primary duration due to diminishing returns. From 1 to 2 s, windows would require twice as much data and doubled prediction latency, and improved by only approximately 0.04 in MCC.

**Table 3:** CNN model “All-task” with different duration.

		“All-task” model				
		ACC	F1	MCC	Specificity	Sensitivity
Model	0.5-s	0.72	0.65	0.42	0.74	0.69
	1-s	0.78	0.73	0.55	0.80	0.75
	2-s	0.8	0.76	0.59	0.79	0.80
	3-s	0.73	0.71	0.47	0.74	0.72

### Feature importance

We analyzed the factors influencing the model predictions by examining feature importance. Feature importance provided insights into which features the machine learning model relied on when making predictions. If pupil diameter was the primary predictor, it suggested that the model used human cognitive reaction for predicting. On the opposite, if the Gaze position was the dominant factor, it indicated that the model’s predictions were influenced more by the task structure itself, like when participants shifted their gaze position toward a specific point on the screen after the ST appeared.

**Fig. 3:**
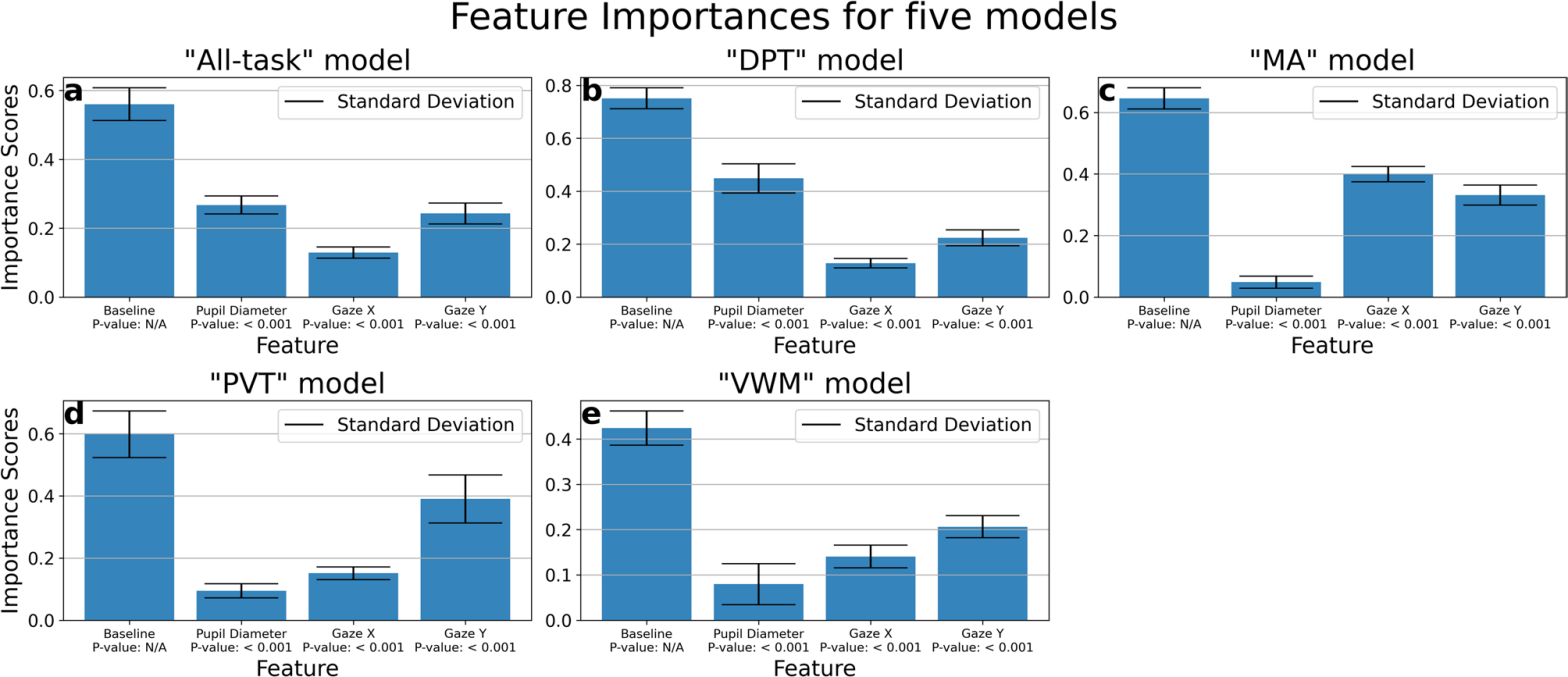
The figure shows feature importance for five models, with each panel corresponding to a specific model. The three features, Pupil Diameter, Gaze X, and Gaze Y, plotted with the performance drop after distortion. The “Baseline” column reports the non-distorted performance for comparison. All values reported in MCC.

Figure 3 displayed feature importance permutation results for five models, using the calculation method mentioned in the “Evaluation metric and feature importance algorithms” section. We performed a T-statistic test between each feature and baseline. All p-values were less than 1E-3. This indicated that all features contributed to the prediction.

In the “MA”, “PVT”, and “VWM” models, we observed that gaze positions (Gaze X and Gaze Y) were the primary factors influencing the models’ predictions. In the “DPT” model, the pupil diameter was most significant contributing feature, with its contribution surpassing that of the other four tasks. Given the PLR effect occurred in the DPT task, the pupil diameter contributed in the DPT task more than to any other tasks, and the model mostly relied on PLR to make predictions. We considered PLR effect was part of the task’s structure because it only appeared in DPT tasks. Therefore, each task-specific model utilized its unique task structure to make predictions.

In the generalized “All-task” model, pupil diameter and gaze position each contributed approximately half of the predictions. In the “Discussion” section, we argued that the generalized model gave more weight to the cognitive reaction factor. For a single task, the task structure would overshadow cognitive reactions, but as the variety of tasks increased, the contribution of human cognitive reactions became more important.

The next question concerned whether the five models could rely solely on cognitive reactions to make predictions, without the task structure. We repeated all the experiments, with the only difference being the removal of gaze position features (Gaze X and Gaze Y). Table 4 had the same setup as Tables 2 and 1 for the CNN model, but without the gaze position features (Gaze X and Gaze Y), only pupil diameter. All performance metrics decreased because of the reduced amount of available data. The “DPT” model was excluded from the comparison due to the PLR effect. The four remaining models showed performance above the random guessing line, indicating that cognitive reactions alone could support prediction. The “All-task” model achieved the highest performance with a decrease of about 0.08 in MCC. The task-specific model “MA”, “PVT”, and “VWM” decreased between 0.13 to 0.24 in MCC without the support of gaze position. In addition, the Pearson correlation coefficient dropped to between 0.5 and 0.6, indicating reduced agreement between the models. These findings indicated that the generalized “All-task” model relied more on cognitive reactions than the specialized models.

**Table 4:** Result for five CNN models using only pupil diameter feature.

		CNN model						
		ACC	F1	MCC	Specificity	Sensitivity	Pearson	McNemar P-value
Model	“All-task”	0.74	0.69	0.47	0.78	0.70	1.0	1.0
	“DPT”	–	–	–	–	–	–	–
	“MA”	0.78	0.53	0.41	0.91	0.44	0.57	< 0.001
	“PVT”	0.77	0.66	0.44	0.85	0.58	0.56	< 0.001
	“VWM”	0.71	0.47	0.30	0.88	0.38	0.51	< 0.001

### Online environment simulation

The previous results were evaluated in an experiment environment, where we had access to the complete dataset, allowing us to segment and evaluate time-series data. However, in real-time scenarios, data was not available at the start of experiments and was less controllable. The online environment required a different approach, where data became available incrementally, and models had to make predictions on the fly. To simulate the online environment, we modified the normalization process. Instead of using the entire dataset for normalization, we used the initial 60 s of data as a baseline and normalized the entire dataset using that baseline. This baseline continued to update as more data became available. For the sampling process, we used a 1-s data window and predicted once every 0.1 s. The remaining methodology was unchanged.

**Fig. 4:**
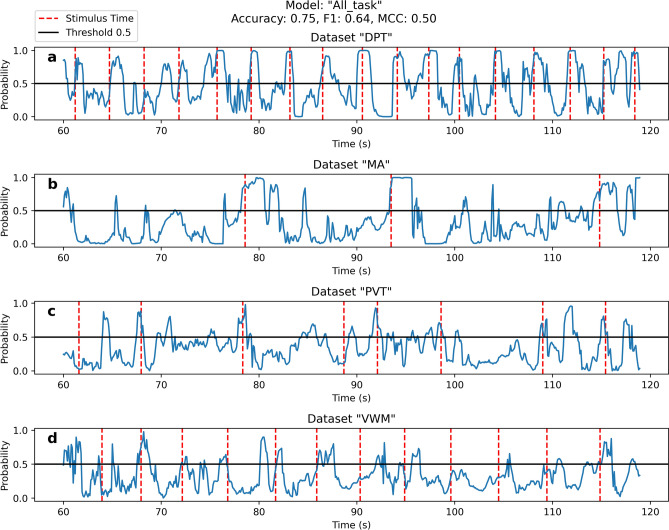
Real-time prediction using the “All-task” model on a single participant. The performance metrics for this participant are displayed in the title. The initial 60 s (0–60 s) are reserved for data normalization, and the subsequent 60 s (60–120 s) display prediction. The red dotted line indicated ST, and the blue line indicates the model’s predicted probability: 1 was ST presence, 0 was ST absence, and values near 0.5 reflect uncertainty in prediction.

Displaying all results would be impractical. Figure 4 showed a sample prediction of the “All-task” model for a 60-s interval for four different datasets from an arbitrary participant. For context, the average performance across participants was: $0.78 \pm 0.05$ in accuracy, $0.73 \pm 0.07$ in F1 score, $0.55 \pm 0.1$ in MCC. The model predicted the majority of ST accurately. The most missing STs were from the “VWM” dataset. This highlights the “VWM” dataset’s lower performance relative to the other three.

In instances where no ST, the model outputted probabilities below 0.5, indicating the absence of cognitive events. Occasionally, we observed peaks in model predictions exceeding the 0.5 threshold, suggesting potential cognitive events. While many of these instances were likely misclassifications, we could not discount the possibility of cognitive events originating outside the trial window or from participant’s internal thoughts. This phenomenon was particularly noticeable in the MA dataset where, after the initial peak corresponding to the ST, we observed secondary peaks approximately 4–5 s later. For example, in the MA dataset panel of Fig. 4, the first ST occurred around 78 s, with the initial peak in predictions around 78–80  s. Then, a second peak was around 82 s. Similar occurrences scattered throughout the MA dataset. The first peak (ST) corresponded to the participant seeing the math problems, and the second peak might relate to the participant attempting to solve them. This suggested the possibility of unrecorded cognitive workloads outside the trial context.

The results for the five models in the online environment: “All-task”, “DPT”, “MA”, “PVT”, and “VWM”, were 0.49, 0.71, 0.60, 0.55, and 0.40 respectively, measured in MCC. Compared to Table 1, all models experienced a decrease of approximately 0.05 in MCC. This illustrates the trade-off between offline and online environments.

## Discussion

To summarize the results, the performance of the five models (“All-task”, “DPT”, “MA”, “PVT”, and “VWM”) were 0.55, 0.75, 0.65, 0.59, and 0.43 measured by MCC respectively. Transitioning from specialization to generalization led to a decrease in specificity while increasing sensitivity. When predicting from a single task, the unique task structure was the primary contributor to the prediction. As more tasks were introduced, the contribution of human cognitive reactions increased. In the online simulation environment, the performance dropped by about 0.05 in MCC across all models.

### Factor influence the model prediction

The unique task structure referred to participants directing their gaze to a specific location when the ST was presented. The PLR effect in DPT was also considered as the structure of the task, as it only appeared in the DPT task. The PLR effect was characterized by a rapid constriction followed by a fast re-dilation of the pupil diameter^33^. The human cognitive reaction was indicated when the pupil diameter increased, reflecting the activation of the sympathetic nervous system which responded to an increase in workload when participants processed ST^31,32^. This increase in pupil diameter was small, on the order of a few tenths of a millimeter^29^.

While training individual tasks, four specialized models relied on unique task structures for prediction. From Table 4, we observed that the model could use human cognitive reaction for prediction, but they relied on task structures. This phenomenon was logical, as task structures were more easily detectable than cognitive reactions. The differences in pupil diameter due to cognitive reactions were minimal, often only a few tenths of a millimeter^29^. It was a weak factor that could be overshadowed by the unique task structure.

In contrast, the “All-task” model needed to find a common pattern across all samples, which was the human cognitive reaction. The human cognitive reaction played a more significant role in the “All-task” model, as evidenced in Fig. 3. While the four task-specific models depended on unique patterns within their respective tasks, the absence of task-type information in the input prevented the model from identifying which sample belonged to which task. As the variety of tasks increased, we expected the “All-task” model to increasingly rely on human cognitive reactions.

#### Brain region activation differences between VWM and DPT, MA, and PVT tasks

In all results, the “VWN” model had the lowest performance with a non-trivial gap. In Table 2, compared to other models, the “VMN” model was on the same specificity level, but its sensitivity was significantly lower. The largest differences between the “VWM” and the other three tasks were attributed to the fundamentally different brain activation regions involved.

The DPT, MA, and PVT tasks engaged different aspects of attention, such as selective attention, sustained attention, and attentional disengagement. In contrast, VWM was a cognitive process that involved storing and manipulating visual information in short-term memory.

These attention-related tasks, like DPT, MA, and PVT, likely involved the anterior cingulate cortex (ACC), a brain region associated with various cognitive functions such as conflict monitoring^34^, error detection^35^, response selection^36^, and executive control^37^. The ACC also regulated emotional processes like affective appraisal and emotional regulation, adapting attention to task demands and emotional context^38^.

VWM, however, might rely more on brain regions like the posterior parietal cortex^39^ and prefrontal cortex^40^, which are involved in encoding, maintaining, and manipulating visual information in a short-term memory buffer. The posterior parietal cortex handled spatial attention and representation^41^, while the prefrontal cortex managed executive control and working memory updating^42^. VWM might not heavily involve the ACC compared to the other tasks, as it did not involve as much conflict, error, or emotional processing.

Differences in cortical activation may influence the strength and timing of pupillary responses, particularly through ACC, which modulates the arousal-related locus coeruleus-norepinephrine (LC-NE) system. According to Adaptive Gain Theory, highly conflicting tasks that involved the ACC triggered activation of the LC-NE system^43^, and the phase of LC-NE activity was shown to predict changes in pupil diameter^44^. Tasks involving the ACC tended to produce large and stereotyped pupil dilation at ST, while tasks like VWM, which rely more on posterior parietal and prefrontal regions than the ACC, might have a weaker or more monotonic change in pupil diameter. This raises the question of whether such differences in brain region activation could directly impact the pupillary responses used for classification. Whether the LC-NE system alone contributed a significant factor to the pupillary signal and altering model performance remained unclear. Further testing and analysis are required to confirm this relationship.

## Limitation

The experiments were conducted in a lab-controlled environment. The online environment simulation was an attempt to replicate real-time scenarios but had limitations because all four tasks were conducted in a lab-controlled setting. Real-life noises and data could not be stimulated. Certain features might not have been as effective outside these environments. For example, gaze position features, where participants focused on specific locations on a screen, might have limitations in real-world settings. In real-world scenarios, points of interest were not the same on a screen. Gaze position was included in the study as it provided information for detecting saccadic and fixation eye movements. We anticipated that in real-world environments, cognitive reactions would be the primary contributing factor, as they reflect cognitive workload and are generalizable. This expectation was supported by the findings from the “All-task” model, which showed that cognitive reaction was an important factor in making predictions.

In addition, because demographic information was not provided, the results could be biased toward specific subgroups and might not have accurately reflected the general population.

## Conclusion

In this study, we developed and evaluated five CNN models using pupillary data to predict stimulus onset times for four different cognitive tasks. We built one generalized model “All-task” and four specialized models. The study highlighted several key findings:

1. The results for the five models (“All-task”, “DPT”, “MA”, “PVT”, and “VWM”) were 0.55, 0.75, 0.65, 0.59, and 0.43, respectively, measured in MCC. When multiple cognitive tasks had been integrated into training, specificity decreased while sensitivity increased. This reflected the trade-off between specialization and generalization.
2. In the specialized models, it was possible to use human cognitive reactions for prediction; however, the models prioritized the unique task structure patterns. As we integrated more cognitive tasks into training, human cognitive reactions contributed more and more to the predictions.
3. In the online environment simulation, the performance of all five models decreased by approximately 0.05 in MCC. This highlighted the trade-off between a dynamic environment and data availability.

For future work, our focus was on enhancing models by incorporating a broader range of participant-specific data. We plan to integrate a wider variety of tasks into training to improve generalization. Furthermore, several known factors influenced pupil diameter, such as body mass^45^, lighting conditions^46^, age^47^, and medication^48^. In future modeling efforts, we intend to incorporate these factors.

The implications of this research had potential applications in real-time detection of cognitively demanding tasks and accurate determination of stimulus onset times, including individual stress level identification. This research could enhance human-computer interaction, improve healthcare systems, develop personalized learning experiences, optimize learning outcomes, and allocate workload based on individual cognitive needs.

## Methodology

### Material

The dataset was part of the Cognitive Resilience and Sleep History research project. Fifty-seven participants volunteered in the research experiment at the University of California, Santa Barbara. No demographic data was provided. The Human Participants Committee of the University of California Santa Barbara (#IRB00000307) and the Army Research Laboratory Human Research Protections Office approved all study procedures. All experiments were conducted in accordance with the approved guidelines and regulations. All participants provided written informed consent.

Each participant followed a standardized protocol, beginning with a relaxation period of 6 min (referred to as “REST”), followed by the completion of four distinct cognitive tasks. Participants could return for up to 10 sessions on separate days (median = 6, SD = 3.33).

Four cognitive tasks were widely recognized cognitive assessments; they were designed to probe various aspects of mental functioning. A brief overview of each task was described below:

**Dot Probe Task (DPT):** This task involved the display of two facial images, each categorized by emotion (angry, happy, or neutral). A subsequent visual probe would appear, and participants asked to identify the probe’s location (left or right). The DPT measured how much faster participants responded to angry stimuli compared to neutral stimuli. DPT is a classic assessment of selective attention (160 trials per session).

**Mental Arithmetic (MA):** Participants solved modular arithmetic problems, with varied in difficulty level (easy and hard). MA tasks were considered a core component of human logical thinking and related to attention, working memory, processing speed, MA ability, and executive function. The difficulty level was presented randomly (40 trials per session).

**Psychomotor Vigilance Task (PVT):** Participants pressed a key when a visual stimulus appeared on the screen. The PVT was a classic measure of sustained attention that measures how participants respond to a simple visual stimulus for an extended period of time (77 trials per session).

**Visual Working Memory (VWM):** Participants memorized a stimulus image and compared it to a second image. The second image could be similar or different. Participants were asked to determine whether the second image matched the first, with difficulty levels varying (1 item for easy level to 6 items for hard level). Each session had 48 trials.

Data was recorded at 250 Hz, capturing pupil diameter, gaze position (Gaze X, Gaze Y). Additionally, behavior data were collected, including the initiation time of each trial (Stimulus Time, ST), and task-specific information. In this paper, ST was used as a primary predictor of cognitive events.

### Sampling method

The goal was to auto-detect cognitive events. ST signified the beginning of each trial, marking new information for participants to process. Therefore, each ST was a cognitive event, and we wanted to predict the location of the ST. The input data had 1-s intervals of three time-series features (Pupil Diameter, Gaze X, and Gaze Y), each recorded at a sampling rate of 250 Hz. The problem was framed as a binary classification task, where the model’s output yielded a probability ranging from 0 (absence of ST) to 1 (presence of ST).

To account for individual differences, we standardized the dataset per participant and session. Participants had different pupil diameters and reacted differently toward ST and PLR. For participants with multiple repeated sessions, we treated each session as a separate entity due to differences in pupil diameter between sessions. These differences may have resulted from factors such as variations in equipment calibration, lighting conditions, or participants’ mental states. To address this, standardization involved calculating the mean and standard deviation of each session, scaling the data to set the mean of data at 0, and the standard deviation at 1. This approach helped minimize the individual variations.

Let the time of ST was at 0 s, for each ST, two samples were generated: sample labeled “0” (absence of ST) from − 1 to 0 s and one sample labeled “1” (presence of ST) from 0.5 to 1.5 s. We chose the start time of 0.5 s to minimize the influence of the PLR that may occur with stimulus presentation^16,46^. Typically, the initial constriction phase of the pupil took about 0.9 to 1-s^33,49^. However, in our dataset, we found that the pupil reached its minimum diameter in approximately 0.6–0.7 s. Since our goal was to capture cognitive responses, we chose to avoid the majority of the constriction phase. In addition, as discussed in “Exploratory data analysis” section, the 0 to 0.5-s window corresponded to saccadic eye movements, during which participants shifted their focus to newly presented information. The pupil dilation occurred after 0.5 s when participants began processing the stimulus. Therefore, we determined that a time frame of 0.5–1.5 s was appropriate to predict cognitive events.

**Fig. 5:**
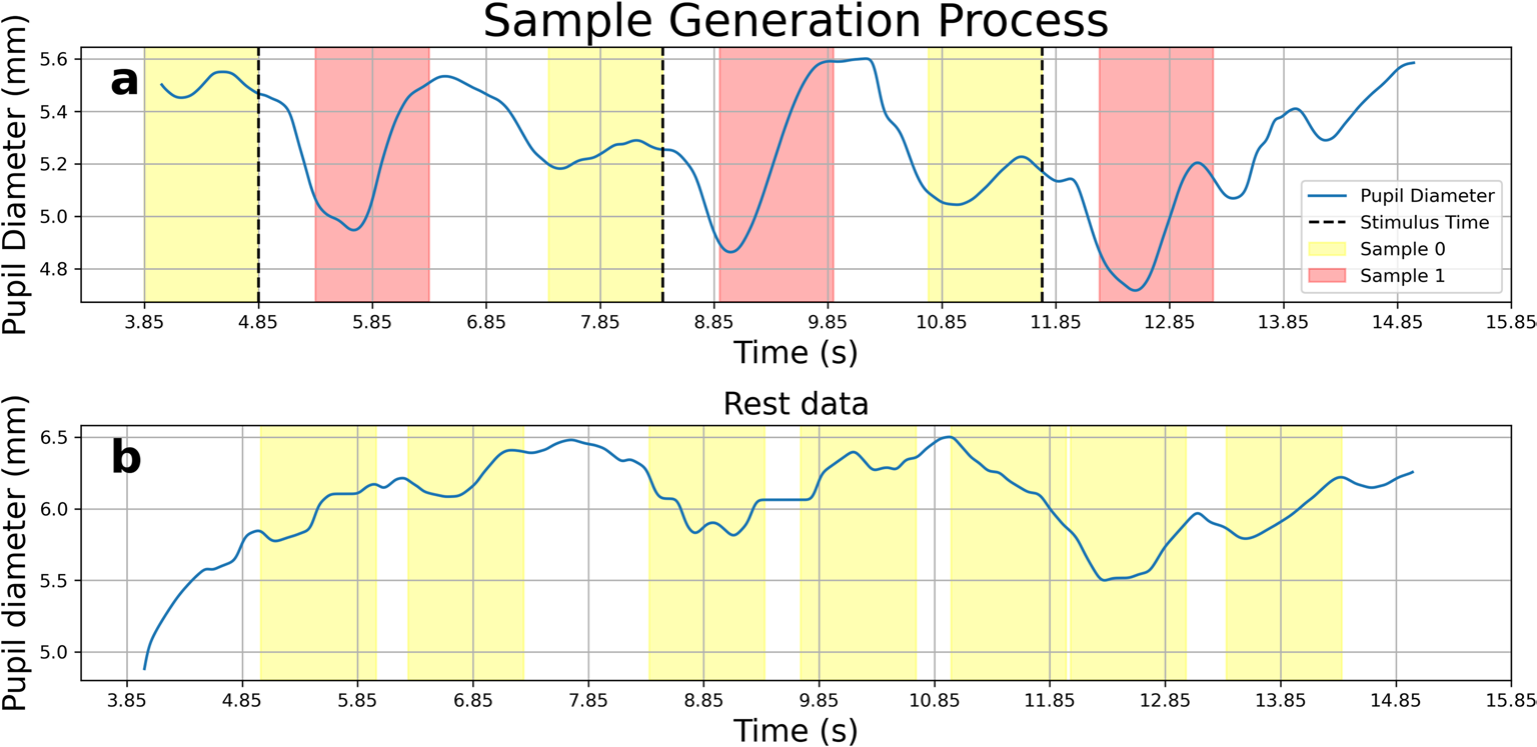
Sample generation process. The panel (**a**) displayed on task data. The black dotted line was the Stimulus Time (ST). For every ST, the generation process generated one “0” sample (yellow) and one “1” sample (red). The panel (**b**) displayed REST data, where non-overlapping “0” samples were randomly generated. Each sample contained 1 s of data.

Next, we randomly selected a 1-s window from “REST” data, and labeled it as a “0” sample. All “REST” data samples were non-overlapping. The process for sample generation was illustrated Fig. 5.

Following data sampling, any missing data samples were removed. Missing data was caused when the eye-tracking devices failed to capture participants’ pupil signals, either because participants looked away from the screen or due to technical errors. After removing invalid samples, the dataset exhibited an imbalance, with “0” class samples being the majority. The percentage for the “1” class was 40%, 34%, 23%, 28%, and 40% for All-task, DPT, MA, PVT, and VWM, receptively. We applied SMOTE (Synthetic Minority Oversampling Technique) to rebalance only the training dataset, while the testing dataset remained unchanged. After SMOTE, the class distribution in the training set was 1:1. This helped the models avoid statistical bias toward either class.

Then, we split the fifty-seven participants into five folds. Two folds had 12 participants each and three folds had 11 participants each. Unlike the standardization process, the splits were made at the participant level rather than at the session level or sample level to avoid data leakage. Splitting by samples could allow samples from the same participant to appear in both the training and testing set, causing data leakage and artificially inflating model performance. After splitting, we applied five-fold cross-validation. In each iteration, three folds were used for training, one fold for validation, and one fold for testing.

### Models architecture

All four model architectures shared a common overall structure and used the same loss function. The distinctions were laid in their specific layer. The layer for each architecture was detailed below, where *x* denoted the input, *b* was the bias and *y* was the output, $\phi$ was the ReLU activation function and *M* was a dropout matrix with a dropout rate of 0.3.

**CNN:** The architecture was a sequential convolutional neural network (CNN). Each layer included a convolutional layer with a ReLU activation function, followed by a max-pooling layer, and a dropout layer. The mathematical representation for each layer was expressed as follows:

1 $$\begin{aligned} y = M \odot P(\phi (W_{conv} * x + b)) \end{aligned}$$

where $W_{conv}$ represented the 1D convolution tensor with a filter size of 5, stride 1, and 64 output channels. *P* was the max-pooling operation with size 2.

**BiLSTM:** The architecture was a bidirectional long short-term memory with each layer that could be expressed in mathematical terms as follows:

2 $$\begin{aligned} y = M \odot [LSTM_f(x);LSTM_b(x)] \end{aligned}$$

where $LSTM_f$ and $LSTM_b$ were forward and backward LSTM layers, each with a hidden dimension of 64.

**RNN:** The architecture was a simple recurrent neural network (RNN). The hidden state update and final output were given by:

3 $$\begin{aligned} \{h_t\}_{t=1}^{250} = \{ M \odot (W_x*x_t + W_h*h_{t-1} + b) \}_{t=1}^{250}, \ \ y = h_{250} \end{aligned}$$

where *t* was the time-step that was recurrent from 1 to 250. $x_t$ and $h_t$ were the input *x* and the hidden state *h* at timestep *t*. *h* had the dimension of 128. $W_x$ and $W_h$ were the weight matrix for the input *x* and hidden state *h*, respectively. The output *y* was equal to $h_{250}$, the hidden state in timestep 250.

**MLP:** The architecture was a multilayer perceptron. Each perceptron layer could be expressed in mathematical terms as follows:

4 $$\begin{aligned} y = M \odot \phi (Wx + b) \end{aligned}$$

*W* was the weight matrix with hidden dimension 128.

All four architectures shared the same general structure and produced two outputs: a classification output ${\hat{Y}}_{clf}$ and a pupil diameter output ${\hat{Y}}_{PD}$. The ${\hat{Y}}_{clf}$ generated a probability score ranging between 0 and 1, where 0 value indicated the absence of ST, and 1 value indicated the presence of ST. It was the main output for optimization. The ${\hat{Y}}_{PD}$ output aimed to reconstruct the pupil diameter from the input sample. It was used solely for review and error-checking purposes. The architecture equation was defined as:

5 $$\begin{aligned} ({\hat{Y}}_{clf}, {\hat{Y}}_{PD}) = W_{l} \cdot P_{ave}(L_4(L_3(L_2(L_1(X)))))) + b_{l} \end{aligned}$$

where *X* was the input sample with three dimensions (N, 250, 3), except for the MLP, which received a reshaped size of (N,750). N was the number of samples, the second dimension was the temporal dimension corresponding to 1 s at 250 Hz, and the third dimension was 3 features (Pupil Diameter, Gaze X, and Gaze Y). Since the MLP could not process temporal sequences directly, the temporal and feature dimensions were merged (i.e., $$250 * 3=750)$$. The layers $$L_1, L_2, L_4, L_4$$ were architecture-specific and differed depending on the model (as described in Eqs. 1–4). The $$P_{ave}$$ referred to the average pooling operation applied exclusively to the temporal dimension, without affecting the other dimensions, to reduce the temporal dimension. This averaging pooling was not applicable in the MLP architecture. The $$W_l$$ and $$b_l$$ were the weight and the bias for the final linear layer. The $${\hat{Y}}_{clf}$$ and $${\hat{Y}}_{PD}$$ were the output of the architecture.

All models trained using the same composite loss function that combined cross-entropy loss, ${\mathcal {L}}_{cel}$ for the classification output ${\hat{Y}}_{clf}$ and *L*1 loss ${\mathcal {L}}_{MAE}$ for the pupil diameter output ${\hat{Y}}_{PD}$. The overall loss function could be expressed as:

6 $$\begin{aligned} {\mathcal {L}} = {\mathcal {L}}_{cel}(\sigma ({\hat{Y}}_{clf}), Y) + \alpha * {\mathcal {L}}_{MAE}(\phi ({\hat{Y}}_{PD}), X_{PD}) \end{aligned}$$

where $\sigma$ and $\phi$ were the Sigmoid and ReLU activation functions respectively. The *Y* was the ground true classification label (either 0 or 1). $\alpha = 0.004$ was a constant scalar, and $X_{PD}$ was the ground true for the input sample containing only the Pupil Diameter feature after removing the Gaze X and Gaze Y features. The ${\hat{Y}}_{PD}$ output was designed for error-checking purposes. If the models were unable to reconstruct the original output from its last layer, it signaled a potential mathematical error within the architecture. However, this should not affect the overall performance of the model. The coefficient $\alpha$ was selected to be small enough to not affect the classification performance, and non-zero to ensure that the model does not neglect the reconstruction task. After testing, we determined that 0.004 was appropriate for the coefficient $\alpha$.

We trained five distinct models for each architecture described above. The key distinction between these models was the training data used. Four models were task-specific, namely “DPT”, “PVT”, “MA”, and “VWM”, trained exclusively on data from their respective tasks. In contrast, the “All-task” model was trained on the entire available training dataset, aiming to evaluate the architecture’s ability to generalize across various tasks.

### Evaluation metric and feature importance algorithms

We selected the Matthews Correlation Coefficient (MCC) as the primary metric. This choice was made because the dataset was imbalanced. MCC was known for its robustness in handling imbalanced datasets^50,51^. Alongside MCC, we included several secondary metrics such as accuracy, F1 score, sensitivity, and specificity.

For the third objective mentioned in the “Introduction” section, we aimed to understand the factors influencing our model’s predictions by performing a feature importance analysis. This analysis helped identify which feature contributed the most weight to the output. We used the Permutation Feature Importance algorithm^52^ to compute feature importance.

Initially, we established a baseline score, denoted $s_{base}$. We evaluated testing samples without any alterations. $s_{base}$ was measured in terms of MCC. Then, we assessed the score for each feature. To accomplish this, we randomly shuffled all data within a specific feature, keeping all other features unchanged. This process allowed us to isolate the impact of the feature under examination. Following this, we conducted predictions and computed the score for these perturbed samples $s_{i}$ anticipating a decrease in performance compared to $s_{base}$. The decrease in performance was quantified and subtracted from $s_{base}$. This iterative process was repeated for all features. To ensure the reliability of these feature importance metrics, we repeated these experiments 100 times $(N = 100)$ and calculated the mean and standard deviation across all features, as expressed in Eq. (7):

7 $$\begin{aligned} I_{j} = \frac{1}{N} \sum _{i=1}^{N}{(s_{base} - s_{i,j})} \end{aligned}$$

where, $I_{j}$ represented the importance score for feature *j*, and $s_{i,j}$ was the MCC score for feature *j* in the *i*-th iteration.

## Data Availability

The data can be provided upon a reasonable request to the corresponding author. The request may subject to approval by the US Army DEVCOM Army Research Laboratory and the Human Participants Committee of the University of California, Santa Barbara.
